# Mediterranean Diet Adherence and Vitamin Intake Adequacy in Spanish University Students: Associations with Body Composition and Physical Activity

**DOI:** 10.3390/nu18040558

**Published:** 2026-02-08

**Authors:** Cristina Petisco-Rodríguez, Gema Barrientos-Vicho, Francisco Javier Alves-Vas, Ignacio Bartolomé Sánchez

**Affiliations:** 1 Faculty of Education, Pontifical University of Salamanca, C/Henry Collet, 52-70, 37007 Salamanca, Spain; cpetiscoro@upsa.es (C.P.-R.); gbarrientosvi@upsa.es (G.B.-V.); fjalvesva@upsa.es (F.J.A.-V.); 2 Faculty of Health Sciences, Pontifical University of Salamanca, 37007 Salamanca, Spain

**Keywords:** Mediterranean diet adherence, dietary assessment, vitamin intake adequacy, young adults, physical activity, body composition

## Abstract

Background/Objectives: This study examined the relationship between adherence to the Mediterranean diet (MD), dietary and vitamin intake, physical activity, and body composition in young adults. Methods: A total of 145 Spanish university students (34 women and 111 men) were included in this cross-sectional study, with a mean body mass index (BMI) of 23 kg/m^2^. MD adherence was assessed using the Mediterranean Diet Adherence Screener (MEDAS). Dietary intake was evaluated through a three-day food record, physical activity using the International Physical Activity Questionnaire (IPAQ), and body composition by bioelectrical impedance analysis. Results: Overall adherence to the MD was moderate. Participants with high MD adherence showed significantly lower body weight (*p* < 0.05; d = 0.4), BMI (*p* < 0.01; d = 0.52), fat mass (*p* < 0.05; d = 0.44), and fat mass percentage (*p* < 0.05; d = 0.38) compared with those with low adherence. Energy (*p* < 0.05; d = 0.41), protein (*p* < 0.05; d = 0.65), and carbohydrate (*p* < 0.05; d = 0.37) intake per kilogram of body weight were higher in the high-adherence group. Fiber intake was greater (*p* < 0.001; d = 0.82) among those with higher MD adherence. Adherence to the MD was also associated with higher intakes of vitamins C (*p* < 0.05; d = 0.39) and E (*p* < 0.05; d = 0.62), retinol equivalents (*p* < 0.05; d = 0.28), and carotenoids (*p* < 0.001; d = 0.79). MD adherence was inversely correlated with body weight (r_s_ = −0.32; *p* < 0.01; r = 0.46) and BMI (r_s_ = −0.34; *p* < 0.01; r = 0.32). Fiber intake showed positive correlations with several water-soluble vitamins, particularly folate (HAG: r_s_ = 0.68; *p* < 0.001; r = 0.81 and LAG: r_s_ = 0.61; *p* < 0.001; r = 0.69). Conclusions: In conclusion, higher adherence to the MD among university students was associated with healthier body composition and improved vitamin intake adequacy. These findings support the promotion of the MD as an effective nutritional strategy to enhance micronutrient intake and overall diet quality in young adults.

## 1. Introduction

The Mediterranean diet (MD) is widely recognized as one of the healthiest dietary patterns and has been consistently associated with reduced all-cause mortality and a lower risk of major chronic non-communicable diseases [1,2,3,4]. Beyond disease prevention, adherence to the MD has been linked to improved cardiometabolic health, reduced systemic inflammation, enhanced insulin sensitivity, and beneficial effects on body weight regulation and adiposity [5,6,7]. These health benefits are largely attributed to the traditional dietary pattern underlying the MD, characterized by a high consumption of vegetables, fruits, legumes, nuts, whole grains, fish, and olive oil, alongside a low intake of red and processed meats and dairy products [8,9]. Consequently, the MD provides a nutrient profile low in saturated and trans fatty acids and cholesterol, while being rich in dietary fiber, complex carbohydrates, monounsaturated fatty acids, vitamins, minerals, and other bioactive compounds, with relatively low energy density [10,11].

Globally, inadequate vitamin intake is most prevalent in vitamin E, riboflavin, and folate [12]. Higher adherence to the MD has been associated with greater intakes of vitamin K [13], improved folate intake and status, and increased levels of vitamins A, C, and B12 [14,15]. Although stronger adherence to the MD has been related to a significantly lower risk of overall micronutrient inadequacy [16,17], vitamin D inadequacy remains common even among adherent populations [18]. Recent evidence indicates that greater adherence to the MD is associated with improved micronutrient intake adequacy and a lower prevalence of vitamin inadequacies compared with Westernized dietary patterns [19]. Moreover, the antioxidant capacity of MD is largely attributable to vitamins C and E and carotenoids, highlighting the importance of dietary diversity within this pattern [20]. Nevertheless, population-based data from the ANIBES study indicate that, despite the traditional MD framework, intakes of fiber, folate, and vitamins A and C remain suboptimal in the Spanish population [21].

Despite its well-documented health benefits, adherence to the MD has declined substantially in Mediterranean countries, particularly among children, adolescents, and young adults [22,23]. This dietary transition is characterized by an increased consumption of energy-dense, nutrient-poor foods, as well as changes in food processing practices that negatively affect nutritional quality [24].

In recent years, the nutritional status of university students has become an issue of considerable concern [25]. Evidence indicates that the majority of university students fail to meet recommended vitamin intake levels, with vitamin E and folate intakes frequently below 50% of the dietary reference intakes [26], and insufficient intakes of vitamins A, C, D, and E commonly reported [27]. Furthermore, adherence to the MD in this population is generally low [28], with only 20–30% of Spanish university students demonstrating good DM adherence [23]. University students constitute a particularly vulnerable group due to the combined effects of academic stress, limited time availability, increased autonomy over food choices, irregular eating patterns and, lack of information about nutritional concepts [29]. In this sense, it has been observed a positive correlation between MD adherence and well-being among university students [30]. Also, limited availability of healthy food options on campus and the perceived high cost of nutritious foods often leads students to prioritize cheap, calorie-dense foods over healthier choices [31]. These dietary behaviors are of concern, as eating habits established during early adulthood tend to persist later in life.

Lifestyle factors, body composition, dietary habits, and physical activity are commonly considered when evaluating MD adherence among university students [23,28,32,33,34,35]. However, to our knowledge, no studies have simultaneously examined MD adherence, overall dietary intake with particular emphasis on vitamin intake adequacy, together with physical activity levels, and body composition within this population. Thus, the present study aimed to investigate the associations between adherence to the MD and the adequacy of overall dietary and vitamin intake in relation to physical activity and body composition among Spanish university students.

## 2. Materials and Methods

### 2.1. Study Design and Participants

A cross-sectional study was conducted among undergraduate students from the Faculties of Education and Health Sciences at the Pontifical University of Salamanca (Spain). The study sample consisted of 145 students enrolled in the Degrees of Physical Activity and Sports Sciences and Human Nutrition and Dietetics. All participants took part in the study on a completely voluntary basis. Data were collected during scheduled class sessions between February and December 2025.

The study protocol was approved by the Ethics Committee of the Pontifical University of Salamanca (Act 17/01/2025) and complied with the ethical principles outlined in the Declaration of Helsinki. Prior to participation, all eligible students received detailed information regarding the study objectives and procedures. Written informed consent was obtained from all participants.

Eligibility criteria included: being an enrolled university student attending on-campus classes, voluntary participation through informed consent, and availability to complete both questionnaires and body composition assessments. Exclusion criteria comprised incomplete or missing data, implausible energy intake values (extremely low or high according to Goldberg calculations [36]), pregnancy or suspected pregnancy, regular medication or specific diet use, acute illness at the time of assessment, and any physical or mental condition that could compromise participation in anthropometric measurements or questionnaire completion.

The preliminary sample size calculation was conducted using the G*Power software (version 3.1.9.6; Heinrich Heine University Düsseldorf, Düsseldorf, Germany, calculated on 7 February 2025). The analysis indicated that a total sample size of 133 participants was required.

A total of 150 students were initially recruited. After excluding Erasmus students and those with incomplete assessments, 145 participants were retained for the final analysis. Although an equal distribution between male and female participants was intended, this was not possible due to the low number of female students to whom access was available.

### 2.2. Instruments

#### 2.2.1. Dietary Assessment

Dietary intake was assessed using an anonymous, web-based questionnaire developed with Google Forms. The questionnaire was administered in person during scheduled class sessions, with supervision and assistance provided by a trained researcher to ensure correct completion. Upon accessing the questionnaire via a QR code, participants were first presented with an information sheet outlining the study objectives and procedures. Electronic informed consent was obtained prior to data collection.

The initial section of the questionnaire gathered sociodemographic information, including age, sex, degree program, and year of study. Dietary intake was subsequently assessed using a three-day food record, consisting of two pre-assigned weekdays and one weekend day. Participants received standardized instructions during class time on how to accurately record food consumption.

Participants reported the type, frequency, and quantity (in grams) of all foods and beverages consumed, covering all main meals, snacks, and drinks consumed between meals. Additional details regarding food preparation and portion size were also collected. The average time required to complete the dietary assessment was approximately 90 min. All these procedures were carried out following the methodology employed by Toro-Román et al. [37,38].

Daily energy and nutrient intakes were calculated based on standardized food composition tables [39] and portion size references. Macronutrient intake (carbohydrates, proteins, and lipids) and micronutrient intake (vitamins) were estimated through ratio conversion performed by the research team using the corresponding food composition data. To improve accuracy and reduce measurement bias, participants were provided with a complementary visual guide illustrating common household measures and food packaging references to facilitate portion size estimation.

Vitamin nutrient density was calculated as the intake of each vitamin per 1000 kcal of total energy intake [40].

Individual nutrient intakes were compared with age- and sex-specific dietary reference intakes (DRIs) established for the Spanish population [39].

#### 2.2.2. Mediterranean Diet Adherence

Adherence to the Mediterranean diet (MD) was assessed using the 14-item Mediterranean Diet Adherence Screener (MEDAS) [41], a tool validated for the Spanish population. The questionnaire includes 12 items assessing the frequency of consumption of key food groups characteristic of the Mediterranean dietary pattern and two items evaluating the type of culinary fat predominantly used and the preference for white versus red meat consumption.

Each item was scored dichotomously (0 = criterion not met; 1 = criterion met), resulting in a total score ranging from 0 to 14 points, with higher scores indicating greater adherence to the MD. In accordance with previously established criteria, participants were classified into two categories of MD adherence: low adherence (<9 points) and high adherence (≥9 points) [34,37].

#### 2.2.3. Anthropometric and Body Composition Measurements

Body weight (kg), basal metabolic rate (kcal), fat mass (kg and %), and lean mass (kg and %) were assessed using bioelectrical impedance analysis with a TANITA BC-418 MA (Tanita Corp., Tokyo, Japan; 0–150 kg, precision 100 g). Standing height was measured to the nearest 0.1 cm using a Seca TM stadiometer (model 213, Hamburg, Germany; 60–210 cm, precision 1 mm).

All anthropometric assessments were conducted in the morning under standardized conditions. Participants were required to fast for at least 8 h (including avoidance of caloric beverages), abstain from caffeine, alcohol, and diuretics, and refrain from vigorous physical activity for at least 12 h prior to measurement. Measurements were performed with participants wearing light clothing and no shoes. Participants were also instructed to empty their bladder immediately before assessment. All measurements were collected by the same trained researcher to minimize inter-observer variability.

Body mass index (BMI) was calculated using Quetelet’s formula [body weight (kg)/height (m^2^)] [42]. BMI was classified according to World Health Organization criteria [43] into normal weight (18.5 ≤ BMI < 25.0 kg/m^2^), overweight (25.0 ≤ BMI < 30.0 kg/m^2^), and obesity (BMI ≥ 30.0 kg/m^2^).

Waist circumference was measured around the abdomen at the level of the umbilicus. Hip circumference was measured at the level of the maximum extension of the buttocks posteriorly in a horizontal plane. Waist to hip ratio was calculated as waist circumference (cm) divided by hip circumference (cm).

#### 2.2.4. Physical Activity Evaluation

Physical activity levels were assessed using the short form of the International Physical Activity Questionnaire (IPAQ-SF) [44]. Participants self-reported the frequency and duration of vigorous-intensity, moderate-intensity, and walking activities performed during the previous seven days.

Total physical activity was calculated and expressed as metabolic equivalent task minutes per week (MET-min/week). The MET score is calculated by multiplying the type of physical activity by the intensity in minutes and days. Based on established IPAQ scoring and classification guidelines, participants were categorized into three physical activity levels: low, moderate, or high.

#### 2.2.5. Statistical Analysis

Statistical analyses were performed using IBM SPSS Statistics version 24.0 for Macintosh^®^. Data distribution was assessed using the Kolmogorov–Smirnov test. As the variables did not meet the assumption of normality, non-parametric tests were applied.

Differences between Mediterranean diet adherence groups were analyzed using the Mann–Whitney U test. Additionally, gender-based differences in the studied variables were examined. Associations between continuous variables were examined using Spearman’s rank correlation coefficient. Data Statistical significance was set at *p* < 0.05. Effect sizes were quantified using Cohen’s d to estimate between-group differences and Cohen’s r to assess the magnitude of correlations. All effect size calculations were conducted in accordance with the methodological guidelines described by Fritz et al. [45] for non-parametric data. Results are reported as medians with interquartile ranges.

## 3. Results

### 3.1. General Characteristics of the Study Population

A total of 145 participants were included in the analysis. University students had a median age of 21.0 (21.0–22.0) years and a median BMI of 23.0 (21.6–25.0) kg/m^2^. The general characteristics of the participants are shown in Table 1. According to BMI classification, most students were classified as normal weight, although a relevant proportion presented overweight.

Sex-specific differences were observed in anthropometric and body composition variables. Men exhibited greater body weight (*p* < 0.01; d = 0.71), BMI (*p* < 0.05; d = 0.09), lean mass (*p* < 0.01; d = 0.81), and basal metabolic rate (*p* < 0.01; d = 0.72), whereas women showed a higher percentage of fat mass (*p* < 0.05; d = 0.37).

With respect to physical activity, men reported higher levels of total (*p* < 0.01; d = 0.45), vigorous (*p* < 0.01; d = 0.37), moderate (*p* < 0.05; d = 0.48), and light (*p* < 0.05; d = 0.08) physical activity, as well as greater daily (*p* < 0.01; d = 0.69) and weekly (*p* < 0.01; d = 0.76) energy expenditure. Energy intake was also higher in men (*p* < 0.05; d = 0.42).

### 3.2. Body Composition and Physical Activity According to Mediterranean Diet Adherence

Comparisons of body composition and physical activity variables between low- and high-adherence groups (High-Adherence Group, HAG, vs. Low-Adherence Group, LAG) to the MD are presented in Table 2. Participants with high adherence to the MD showed significantly lower body weight (*p* < 0.05; d = 0.4) and BMI (*p* < 0.01; d = 0.52) compared with those with low adherence.

In addition, the LAG presented significantly higher fat mass, both in absolute terms (*p* < 0.05; d = 0.44) and as a percentage of total body weight (*p* < 0.05; d = 0.38). No statistically significant differences were observed between groups for physical activity variables or energy expenditure, including total, moderate, vigorous, and light physical activity.

### 3.3. Dietary Intake According to Mediterranean Diet Adherence

Macro and micronutrient intake according to MD adherence is shown in Table 3. Although total daily energy intake did not differ significantly between groups, participants with high adherence exhibited higher energy intake per kilogram of body weight (*p* < 0.05; d = 0.40). Although the daily reference values presented in Table 3 are reported in a general manner, mean daily energy requirements differ between males (3000 Kcal/day) and females (2300 Kcal/day).

High adherence to the MD was associated with a significantly higher intake of protein (*p* < 0.05; d = 0.65) and carbohydrates (*p* < 0.05; d = 0.37) adjusted for body weight, as well as a markedly higher fiber intake (*p* < 0.001; d = 0.82). No significant differences were observed in total lipid intake; however, the high-adherence group showed a more favorable fatty acid profile, including a lower ω-6/ω-3 ratio (*p* < 0.05; d = 0.73).

Regarding micronutrients, participants with high adherence to the MD showed significantly higher intakes of vitamin C (*p* < 0.05; d = 0.39), vitamin E (*p* < 0.05; d = 0.62), retinol equivalents (*p* < 0.05; d = 0.28), and carotenoids (*p* < 0.001; d = 0.79).

### 3.4. Vitamin Density and Nutritional Adequacy

To minimize the potential confounding effect of total energy intake, vitamin intakes were also expressed as nutrient density per 1000 kcal (Table 4). Students with high MD adherence showed significantly higher densities of ascorbic acid (*p* < 0.05; d = 0.32), retinol equivalents (*p* < 0.05; d = 0.66), retinol (*p* < 0.05; d = 0.74), carotenes (*p* < 0.001; d = 0.81) and, vitamin E (*p* < 0.01; d = 0.48), compared with those with LAG. These results indicate that the higher micronutrient intake observed among students with greater MD adherence was attributable to improved dietary quality rather than higher caloric intake.

### 3.5. Associations Between Mediterranean Diet Adherence, Body Composition, and Physical Activity

Correlations between MD adherence score and body composition and physical activity variables are presented in Table 5. In the total sample, MD adherence was negatively correlated with body weight (r_s_ = −0.32; *p* < 0.01; r = 0.46) and BMI (r_s_ = −0.34; *p* < 0.01; r = 0.32), as well as with absolute lean mass (r_s_ = −0.29; *p* < 0.01; r = 0.38).

In men, adherence to the MD was negatively associated with weight (r_s_ = −0.24; *p* < 0.05) BMI (r_s_ = −0.27; *p* < 0.05; r = 0.29), fat mass (r_s_ = −0.29; *p* < 0.05; r = 0.4) lean mass (r_s_ = −0.18; *p* < 0.05; r = 0.35) and lean percentage (r_s_ = −0.22; *p* < 0.05; r = 19). No significant correlations were found between MD adherence and physical activity variables in the total sample or when stratified by sex.

### 3.6. Associations Between Fiber Intake and Water-Soluble Vitamins

The correlation analysis between daily fiber and water-soluble vitamin intakes is presented in Table 6. In the high-adherence group, fiber intake was positively correlated with thiamine (r_s_ = 0.52; *p* < 0.001; r = 0.65), riboflavin (r_s_ = 0.49; *p* < 0.001; r = 0.69), niacin (r_s_ = 0.35; *p* < 0.05; r = 0.41), pyridoxine (r_s_ = 0.46; *p* < 0.001; r = 0.66), folic acid (r_s_ = 0.68; *p* < 0.001; r = 0.55), cobalamin (r_s_ = 0.37; *p* < 0.05; r = 0.4), and ascorbic acid (r_s_ = 0.32; *p* < 0.05; r = 0.48). In contrast, fewer and weaker correlations were observed in the LAG. Notably, fiber intake was positively correlated with folate intake in the overall sample and within MD adherence groups (HAG: r_s_ = 0.68; *p* < 0.001; r = 0.81 and LAG: r_s_ = 0.61; *p* < 0.001; r = 0.69).

## 4. Discussion

The present study examined the level of adherence to the MD among young university students and explored whether different levels of adherence were associated with physical activity levels, body composition, and the nutritional composition and quality of the diet, as well as other quantitative aspects related to dietary patterns.

The study included a sample of 145 young university students, predominantly male. This sex imbalance reflects the enrollment characteristics of the degree programs from which participants were recruited and has been reported in previous studies conducted in similar academic contexts. Importantly, the sample size is comparable to [27] or exceeds [35] that of other studies assessing MD adherence and related variables, supporting the robustness of the present findings.

Regarding anthropometric characteristics, the LAG body composition results may indicate that lower adherence to the MD is associated with a less favorable body composition profile, characterized by higher fat mass, as it has been previously reported [46,47]. In line with previous studies conducted in young adult and university populations, higher adherence to the MD tends to be associated with more favorable body composition outcomes, including lower adiposity and healthier weight status distributions [35]. Although the differences observed in BMI categories did not reach statistical significance, the HAG presented a higher proportion of individuals with normal weight (72.7% vs. 66.7%) and underweight status (8.9% vs. 0%), as well as a lower prevalence of overweight (19.0% vs. 28.8%). Notably, no cases of obesity were observed in the HAG, whereas 4.5% of participants in the LAG were classified as obese.

It should be highlighted that adiposity and overweight are conditions with a multifactorial etiology, influenced by both dietary patterns [48] and physical activity levels [49]. In this context, no significant differences were observed between groups in variables related to physical activity habits, energy expenditure, or basal metabolic rate.

Therefore, while causality cannot be established due to the cross-sectional design, the observed differences in body composition in this population are more likely to be related to dietary habits, particularly adherence to the MD, rather than to differences in physical activity or energy metabolism.

The quantitative analysis of dietary habits, presented in Table 3, revealed notable differences in the nutritional composition of the diets followed by participants with different levels of MD adherence. The HAG exhibited a higher total energy and fiber intake as well as higher protein intake relative to body weight.

Daily energy intake is well recognized as a key determinant of adiposity indices and overall body composition [50]. However, despite a higher caloric intake and comparable levels of physical activity relative to the LAG, the HAG demonstrated more favorable body composition outcomes. This apparent paradox may be partially explained by qualitative aspects of the diet, including higher fiber and protein intake.

First, the thermogenic effects associated with protein digestion and metabolism are well established [51], and adequate protein intake relative to body weight is considered a key determinant in dietary strategies aimed at weight loss and/or body recomposition [52]. In this regard, a protein intake of approximately 1.6 g·kg^−1^·day^−1^ has been proposed as a minimum recommended threshold to support these outcomes [53]. This intake level was consistent with the mean values observed in the HAG, whereas participants in the LAG exhibited significantly lower protein intakes relative to body weight (g/kg/day).

In addition, dietary fiber intake represents another nutritional parameter of considerable metabolic relevance [54]. Fiber slows gastric emptying and nutrient absorption, thereby contributing to the regulation of glycemic responses [55] and postprandial insulin excursions [56]. Through these mechanisms, higher fiber intake may favor improved energy metabolism, substrate oxidation and lipid utilization [57]. These physiological processes have been linked to adiposity and overweight status and could therefore partly account for the more favorable body composition outcomes observed in the HAG. Consistent with these findings, adherence to the MD has been associated in previous studies with enhanced insulin sensitivity and improved regulation of body weight and adiposity [5,6,7].

With respect to vitamin intake, it is well recognized that fiber-rich foods constitute an important dietary source of several vitamins [58]. In this context, differences between groups were observed in the intake of ascorbic acid, retinol equivalents, carotenoids, and vitamin E, with generally higher values in the HAG.

To allow for a more meaningful comparison of dietary quality, nutrient density was assessed by calculating vitamin content per 1000 kcal consumed [40]. As shown in Table 4, this analysis corroborated the aforementioned trends, indicating that diets characterized by higher adherence to the MD tend to exhibit greater micronutrient density.

While causality cannot be inferred, this higher nutritional density may partially contribute to the favorable metabolic and health-related profiles associated with greater adherence to the Mediterranean dietary pattern.

In addition, the correlation between dietary fiber intake and vitamin intake was examined in both adherence groups. As shown in Table 6, the LAG exhibited several statistically significant but generally weak correlations. In contrast, the HAG showed positive correlations across all vitamins analyzed, with greater statistical strength.

Collectively, these findings indicate that higher adherence to the MD is associated with greater fiber intake, which in turns shows consistent and stronger correlations with the intake of water-soluble vitamins, particularly folate. Furthermore, diets with greater adherence to the Mediterranean dietary pattern tend to exhibit both higher absolute daily intakes and greater nutrient density of several of these vitamins. While these associations do not imply causality, they support the notion that overall dietary quality improves with increasing adherence to the MD.

Finally, it has been previously indicated that previous studies have reported an association between adherence to the MD and more favorable levels of body adiposity [5,7]. In the present study, as shown in Table 5, a modest negative correlation was observed between MD adherence and both body weight and BMI. These associations were observed exclusively in male participants.

Additionally, among men, MD adherence was negatively correlated with body fat percentage, as well as with lean mass and lean mass percentage. Although the magnitude of these correlations was modest, these findings are generally consistent with existing literature and suggest that higher adherence to the Mediterranean dietary pattern may be associated with more favorable body composition profiles [5,6,7], particularly in male university students. However, given the cross-sectional nature of the study, these associations should be interpreted with caution.

The inverse association between adherence to the Mediterranean Diet and lean mass observed in men should be interpreted with particular caution. This observation may, at least in part, be related to the fact that neither adherence group achieved the recommended energy intake, given that hypocaloric dietary patterns or energy-restricted nutritional states have been associated with reductions in body weight, as well as in both fat and lean mass [59]. Furthermore, this association may also reflect the influence of unmeasured lifestyle- or behavior-related factors, and the potential contribution of residual confounding cannot be excluded.

Overall, these findings are consistent with previous evidence [34,60] and reinforce the role of the MD as a marker of higher dietary quality and micronutrient adequacy in young adults. These results highlight the importance of promoting Mediterranean dietary patterns during university years to support adequate vitamin intake and long-term health [61]. To enhance vitamin intake, nutrient density, and overall diet quality, this population should increase the consumption of selected vitamin-rich food groups. Adequate ascorbic acid intake may be achieved through the regular consumption of vegetables such as broccoli, tomatoes, and peppers, as well as fruits including citrus fruits and kiwifruit [62]. With respect to retinol intake, animal-derived foods such as liver, eggs, white and oily fish, crustaceans, and other seafood represent important dietary sources [63]. Furthermore, the intake of carotenoids may be increased through the consumption of vegetables such as pumpkin, carrots, spinach, and chard, in addition to fruits including mango, persimmon, and plums [63]. Finally, to improve vitamin E intake, the consumption of nuts (e.g., hazelnuts and almonds), seeds (e.g., flaxseed), legumes (e.g., soybeans), and vegetable oils, particularly olive oil and flaxseed oil, is recommended [62].

The comparatively high adherence to the MD observed may, at least in part, be related to the geographical and cultural context of the study, which was carried out in Castilla y León (Spain), a region with dietary traditions closely aligned with the Mediterranean dietary pattern.

Several limitations of this study warrant consideration. First, the cross-sectional design inherently limits the ability to establish causal relationships between MD adherence, vitamin intake, body composition, and lifestyle-related factors. Second, dietary intake was evaluated using self-reported three-day food records, a method that is susceptible to recall bias and misreporting. Although widely accepted and suitable for detailed nutrient analysis, this approach may not accurately reflect long-term habitual dietary patterns. Third, the study sample comprised university students enrolled in health-related degree programs, potentially limiting the external validity and generalizability of the findings to the wider university population. Furthermore, the relatively small representation of students from the Human Nutrition and Dietetics program reduced the statistical power of subgroup analyses by academic discipline. The marked predominance of male participants may further constrain the applicability of the results to female university students. Finally, vitamin intake was assessed exclusively through dietary data rather than biochemical biomarkers, which may not fully capture true micronutrient status. Notwithstanding these limitations, the present study provides novel and meaningful insights into the associations between MD adherence, vitamin intake adequacy, and body composition among young adults.

Another important limitation of this study is the potential bias associated with self-administered dietary records [64]. To mitigate this source of error, participants received standardized instructions regarding the timing and procedures for weighing foods and were trained to accurately estimate portion sizes and corresponding weights of commonly consumed foods using images and practical examples representative of the country’s dietary patterns. A related limitation concerns the reliability of the nutritional analysis tools employed. Although these tools are not entirely free of limitations, they have been widely used in previous research to assess intakes of both essential micronutrients [39,65] and potentially toxic elements [37,66]. Moreover, this methodological approach is increasingly recognized as a valuable tool in both clinical and professional practice [67].

Finally, caution should be exercised when generalizing the findings of the present study. Although the sample size exceeded the minimum required, the study population was highly specific and showed a notable imbalance in sex distribution (111 men vs. 34 women). Consequently, to enhance the external validity of the results, future research should include larger samples and incorporate additional population groups, such as adults and older individuals, as well as a more balanced distribution between sexes.

Future research should therefore prioritize more balanced sex representation to enable the investigation of potential sex-specific differences in body composition and physical activity behaviors. Also, expanding the study to other academic disciplines and institutions using longitudinal designs would enhance the generalizability and robustness of the findings.

## 5. Conclusions

In conclusion, adherence to the MD was associated with significant differences in dietary composition, including energy, carbohydrate, and protein intake relative to body weight. Notable differences were observed in fiber intake, as well as in carotenoids and other vitamins, which were also reflected in a higher nutrient density of the diet among individuals with high adherence.

Fiber intake emerged as a key factor, showing strong and significant correlations within the HAD. Furthermore, higher adherence to the MD was associated with more favorable weight, BMI, and adiposity indices. Overall, these findings support the beneficial role of the MD in nutritional quality and body composition.

## Figures and Tables

**Table 1:** General characteristics of the participants.

	All (n = 145)	Men (n = 111)	Women (n = 34)
Age (years)	21.00 (21.00–22.00)	21.00 (21.00–22.00)	20.00 (18.75–21.00)
Height (m)	1.74 (1.69–1.81)	1.77 (1.73–1.83) *^●^	1.61 (1.58–1.68)
Weight (kg)	71.60 (61.15–80.00)	75.00 (68.50–83.00) **^●●●^	57.50 (51.97–63.00)
BMI (kg/m^2^)	23.04 (21.55–25.02)	23.70 (22.00–25.53) *^●^	22.00 (20.23–22.87)
Obesity (n, %)	n = 3 (2.2%)	n = 3 (2.7%)	n = 0 (0%)
Overweight (n, %)	n = 34 (23.4%)	n = 30 (27.9%)	n = 3 (8.8%)
Normal weight (n, %)	n = 101 (69.6%)	n = 74 (66.7%)	n = 30 (88.3%)
Underweight (n, %)	n = 7 (4.8%)	n = 3 (2.7%)	n = 1 (2.9%)
WHR (cm)	0.80 (0.76–0.84)	0.81 (0.78–0.84)	0.75 (0.70–0.80)
≤0.85 (n, %)	n = 121 (83.4%)	n = 88 (79.3%)	n = 33 (97.1%)
>0.85 (n, %)	n = 24 (16.6%)	n = 23 (20.7%)	n = 1 (2.9%)
Fat Mass (kg)	11.20 (8.50–14.60)	10.40 (8.05–14.10) *^●●^	14.65 (11.20–16.85)
Fat Mass (%)	15.10 (11.20–18.40)	14.00 (11.00–17.20) *^●●^	24.90 (19.62–27.72)
Lean Mass (kg)	62.30 (56.90–69.70)	64.20 (59.65–70.15) **^●●●^	44.50 (41.25–47.15)
Lean Mass (%)	84.40 (81.53–88.08)	85.46 (82.68–88.74) *^●●^	75.09 (72.64–79.34)
Basal Metabolism (Kcal/day)	1826 (1611–2069)	1901 (1741–2120) **^●●●^	1441 (1325–1602)
Daily Physical Activity (hours/day)	2.11 (1.46–3.07)	2.23 (1.66–3.14)	1.60 (0.82–2.62)
Total Physical Activity (hours/week)	14.50 (10.25–20.00)	15.00 (11.66–21.00) **^●●^	11.00 (5.08–16.75)
Intense Activity (hours/week)	5.33 (3.00–8.66)	6.00 (3.33–9.00) **^●●^	2.83 (0.00–6.00)
Moderate Activity (hours/week)	2.00 (0.91–6.00)	3.00 (1.00–6.00) *^●●^	2.00 (0.00–4.00)
Light Activity (hours/week)	5.25 (3.00–10.50)	6.67 (3.50–11.67) *^●^	3.75 (2.25–8.04)
Daily Energy Expenditure (METmin/day)	686.55 (441.96–954.64)	731.35 (511.74–1015.71) **^●●^	495.14 (306.14–743.78)
Weekly Energy Expenditure (METmin/week)	4294 (2772–5763)	4805 (3436–6288) **^●●^	3039 (1786–4756)
Energy Intake (Kcal/day)	2028.85 (1653.59–2582.14)	2099.13 (1678.80–2644.58)	1866.27 (1207.89–2140.07)
Energy Intake (kJ/day)	7984.41 (6283.13–10,294.33)	8255.48 (6882.80–10,461.25) *^●●^	7178.04 (4875.24–8884.43)
Protein Intake (g/day)	104.95 (82.49–136.45)	106.23 (84.64–146.01)	96.00 (59.88–111.97)
Lipid Intake (g/day)	75.12 (58.55–107.50)	81.02 (60.24–109.04)	68.67 (49.55–88.62)
Carbohydrate Intake (g/day)	227.74 (168.99–310.13)	235.60 (183.44–319.32)	202.86 (137.47–276.16)
Fiber Intake (g/day)	12.40 (6.89–20.54)	11.96 (6.31–20.48)	12.46 (7.83–21.34)

Kcal: Kilocalorie; METmin: Metabolic Equivalent of Task minutes; BMI: Body Mass Index; WHR: Waist to Hip Ratio. Differences between sex: * *p* < 0.05; ** *p* < 0.01; Effect Size (d): ^●^ low < 0.1; ^●●^ medium 0.11–0.49; ^●●●^ large > 0.5.

**Table 2:** Comparison of body composition and physical activity characteristics of low- and high-adherence groups to Mediterranean Diet.

	High-Adherence Group (n = 79)	Low-Adherence Group (n = 66)
Men (n, %)	n = 57 (72.2%)	n = 54 (81.8%)
Women (n, %)	n = 22 (27.8%)	n = 12 (18.2%)
Age (years)	21.00 (21.00–22.00)	21.00 (21.00–22.00)
Height (m)	1.74 (1.68–1.80)	1.74 (1.69–1.82)
Weight (kg)	68.40 (60.00–78.00)	73.65 (66.05–82.00) *^●●^
BMI (kg/m^2^)	22.70 (20.90–24.30)	23.90 (22.17–25.67) **^●●●^
Obesity (n, %)	n = 0 (0%)	n = 3 (4.5%)
Overweight (n, %)	n = 15 (19%)	n = 19 (28.8%)
Normal weight (n, %)	n = 57 (72.2%)	n = 44 (66.7%)
Underweight (n, %)	n = 7 (8.9%)	n = 0 (0%)
WHR (cm)	0.79 (0.75–0.83)	0.81 (0.77–0.84)
≤0.85 (n, %)	n = 64 (81.0%)	n = 57 (86.4%)
>0.85 (n, %)	n = 15 (19.0%)	n = 9 (13.6%)
Fat Mass (kg)	10.10 (7.30–13.80)	12.10 (9.17–14.70) *^●●^
Fat Mass (%)	14.00 (10.80–17.65)	16.30 (12.35–18.72) *^●●^
Lean Mass (kg)	60.90 (54.00–69.50)	64.65 (59.45–69.92)
Lean Mass (%)	85.36 (81.62–88.81)	83.71 (81.42–87.62)
Basal Metabolism (Kcal)	1795.00 (1592.00–2043.00)	1882.50 (1672.25–2081.75)
Daily Physical Activity (hours/day)	2.04 (1.48–3.19)	2.21 (1.22–2.87)
Total Physical Activity (hours/week)	14.00 (10.31–20.75)	14.87 (8.22–19.08)
Intense Activity (hours/week)	6.00 (3.00–10.00)	4.75 (1.00–7.50)
Moderate Activity (hours/week)	2.00 (1.00–6.67)	2.25 (0.56–4.75)
Light Activity (hours/week)	5.25 (2.33–11.67)	5.54 (3.45–10.50)
Daily Energy Expenditure (METmin/day)	693.25 (495.14–965.57)	679.85 (423.71–874.50)
Weekly Energy Expenditure (METmin/week)	4294.00 (3057.00–6223.00)	4372.50 (2583–5592)

Kcal: Kilocalorie; METmin: Metabolic Equivalent of Task minutes; BMI: Body Mass Index; WHR: Waist to Hip Ratio; Differences between groups: * *p* < 0.05; ** *p* < 0.01. Effect Size (d): ^●●^ medium 0.11–0.49; ^●●●^ large > 0.5.

**Table 3:** Comparison of macro and micronutrients intake of low- and high-adherence groups to Mediterranean Diet.

		High-Adherence Group (n = 79)		Low-Adherence Group (n = 66)	
	DRI	Value	% DRI	Value	% DRI
Energy Intake (Kcal/day)	3000	2116.74 (1695.82–2710.53)	81	2013.73 (1574.08–2484.64)	76
Energy Intake (Kcal/kg/day)	-	30.87 (23.82–41.07)	-	27.02 (20.38–34.53) *^●●^	-
Energy Intake (kJ/day)	12,552	8143.78 (6672.15–10,738.84)	83	7784 (6171–9903)	74
Energy Intake (kJ/Kg/day)	-	123.38 (93.24–163.40)	-	107.43 (80.10–137.50) *^●●^	-
Protein Intake (g/day)	86	106.44 (84.89–136.24)	123	103.60 (77.50–139.33)	120
Protein Intake (g/kg/day)	-	1.60 (1.19–2.05)	-	1.32 (1.05–1.89) *^●●●^	-
Lipid Intake (g/day)	76	75.02 (57.02–107.23)	98	77.58 (59.79–107.94)	102
Lipid Intake (g/kg/day)	-	1.22 (0.82–1.55)	-	1.08 (0.73–1.47)	-
SFA	<25	24.19 (16.28–32.56)	96	24.35 (16.96–33.51)	97
MUFA	51	29.92 (20.24–41.54)	58	27.80 (19.37–43.14)	55
PUFA	13	9.10 (5.50–14.22)	70	8.23 (5.91–14.58)	63 *^●●^
TUFA	64	39.91 (27.11–57.09)	62	40.47 (27.87–57.46)	63
SFA/TUFA Ratio	≥2	1.67 (1.34–2.34)	83	1.66 (1.26–2.06)	83
ω-6/ω-3 Ratio	1–4	2.59 (1.65–8.6)	38	4.12 (1.56–9.12) *^●●●^	27 *^●●●^
Cholesterol	<300	419.71 (243.88–619.46)	66	431.52 (277.14–635.57)	52 *^●●^
Carbohydrate Intake (g/day)	316	252.44 (181.59–315.13)	80	206.94 (151.10–290.16)	65 *^●●^
Carbohydrate Intake (g/kg/day)	-	3.60 (2.67–4.85)	-	2.98 (2.11–4.29) *^●●^	-
Fiber Intake (g/day)	25	14.71 (8.2–25.03)	59	9.31 (5.56–15.38) ***^●●●^	37 ***^●●●^
Thiamine (mg/day)	1.2	1.46 (0.94–1.98)	122	1.36 (0.87–2.07)	113
Riboflavin (mg/day)	1.8	1.49 (1.25–2.15)	83	1.49 (1.11–2.14)	82
Niacin (mg/day)	20	38.30 (29.61–57.61)	115	39.53 (29.20–53.85)	116
Pyridoxine (mg/day)	1.8	1.50 (1.09–2.47)	83	1.58 (1.20–2.37)	88
Folic Acid (µg/day)	400	133.15 (95.36–223.14)	33	126.57 (77.24–174.67)	31
Cobalamin (µg/day)	2	5.06 (3.16–7.07)	253	4.85 (3.22–7.45)	242
Ascorbic Acid (mg/day)	60	80.12 (42.15–148.86)	133	60.14 (28.52–98.62) *^●●^	100 *^●●^
Retinol Equivalents (µg/day)	1000	480.00 (268.81–621.01)	48	329.57 (220.90–526.04) *^●●^	32
Retinol (µg/day)	1000	204.99 (111.96–286.56)	21	174.58 (117.41–289.23)	17
Carotenes (µg/day)	750	1122.59 (542.50–2135.16)	149	469.48 (175.20–1093.60) ***^●●●^	62 ***^●●●^
Calciferol (µg/day)	15	2.88 (0.70–10.48)	38	3.15 (0.91–12.64)	42
Vitamin E (mg/day)	12	4.05 (2.18–6.19)	33	2.81 (1.40–5.17) *^●●●^	23 *^●●^
Vitamin K (µg/day)	90	66.40 (35.14–87.00)	67	70.02 (42.12–91.80)	69

Kcal: Kilocalorie; DRI: Dietary Recommended Intake; % DRI: disparity between reported consumption and the level needed for adequacy; SFA: Saturated Fatty Acid; MUFA: Monounsaturated Fatty Acid; PUFA: Polyunsaturated Fatty Acid; TUFA: Total Unsaturated Fatty Acid; ω: omega; Differences between groups: * *p* < 0.05; *** *p* < 0.001. Effect Size (d): ^●●^ medium 0.11–0.49; ^●●●^ large > 0.5.

**Table 4:** Comparison of nutritional density of vitamins between low- and high-adherence groups to Mediterranean Diet.

	All (n = 145)	High-Adherence Group (n = 79)	Low-Adherence Group (n = 66)
Thiamine (mg/1000 Kcal)	0.75 (0.56–0.91)	0.76 (0.57–0.92)	0.73 (0.56–0.90)
Riboflavin (mg/1000 Kcal)	0.78 (0.63–0.95)	0.77 (0.62–0.94)	0.78 (0.65–0.97)
Niacin (mg/1000 Kcal)	18.76 (15.49–25.65)	18.72 (15.16–23.41)	18.81 (15.98–26.57)
Pyridoxine (mg/1000 Kcal)	0.79 (0.64–1.01)	0.77 (0.60–1.05)	0.81 (0.66–1.02)
Folic Acid (µg/1000 Kcal)	64.36 (50.67–84.18)	64.28 (53.63–87.52)	64.43 (43.66–83.11)
Cobalamin (µg/1000 Kcal)	2.52 (1.63–3.32)	2.53 (1.61–3.31)	2.48 (1.66–3.34)
Ascorbic Acid (mg/1000 Kcal)	37.05 (19.51–55.24)	41.08 (25.86–59.39)	30.61 (18.37–55.12) *^●●^
Retinol Equivalents (µg/1000 Kcal)	200.02 (124.57–277.82)	220.29 (147.91–308.49)	163.66 (114.94–255.00) *^●●●^
Retinol (µg/1000 Kcal)	88.54 (64.98–128.72)	89.74 (64.96–129.05)	85.39 (63.26–127.84) *^●●^
Carotenes (µg/1000 Kcal)	433.44 (143.77–909.35)	553.14 (265.88–1057.99)	226.62 (95.70–577.45) ***^●●●^
Calciferol (µg/1000 Kcal)	1.34 (0.44–4.92)	1.22 (0.40–4.46)	1.39 (0.48–5.60)
Vitamin E (mg/1000 Kcal)	1.71 (1.06–2.68)	1.88 (1.20–2.83)	1.43 (0.80–2.29) **^●●^
Vitamin K (µg /1000 Kcal)	41.15 (35.88–64.19)	45.77 (38.47–69.95)	43.66 (21.00–65.79)

Kcal: Kilocalorie; Differences between groups: * *p* < 0.05; ** *p* < 0.01; *** *p* < 0.001. Effect Size (d): ^●●^ medium 0.11–0.49; ^●●●^ large > 0.5.

**Table 5:** Correlation study of the level of adherence to Mediterranean diet and body composition and physical activity variables.

	All (n = 145)	Men (n = 111)	Women (n = 34)
Weight (kg)	−0.32 **^●●^	−0.24 *^●^	−0.18
BMI (kg/m^2^)	−0.34 **^●●^	−0.27 *^●^	−0.3
WHR (cm)	−0.13	−0.09	−0.12
Fat Mass (kg)	−0.18	−0.15	0.02
Fat Mass (%)	−0.09	−0.29 *^●●^	−0.09
Lean Mass (kg)	−0.29 *^●●^	−0.24 *^●●^	0.28
Lean Mass (%)	−0.19	−0.27 *^●●^	0.12
Basal Metabolism (Kcal/day)	−0.62	−0.49	0.12
Daily Physical Activity (hours/day)	0.31	0.11	−0.12
Total Physical Activity (hours/week)	0.05	0.09	−0.08
Intense Activity (hours/week)	0.17	0.22	0.08
Moderate Activity (hours/week)	0.11	0.19	−0.19
Light Activity (hours/week)	0.03	0.15	−0.27
Daily Energy Expenditure (METmin/day)	0.18	0.16	−0.12
Weekly Energy Expenditure (METmin/week)	0.09	0.14	0.05

Kcal: Kilocalorie; METmin: Metabolic Equivalent of Task minutes; Spearman’s rank correlation coefficient (r_s_): * *p* < 0.05; ** *p* < 0.01. Effect Size (r): ^●^ low < 0.1; ^●●^ medium 0.11–0.49.

**Table 6:** Correlation study of the daily fiber intake and the intake of water-soluble vitamins.

	High-Adherence Group (n = 79)	Low-Adherence Group (n = 66)
Thiamine (B1)	0.52 ***^●●●^	0.27
Riboflavin (B2)	0.49 ***^●●●^	0.22 *
Niacin (B3)	0.35 *^●●^	0.31 *
Pyridoxine (B6)	0.46 ***^●●●^	0.33 *^●●^
Folic Acid (B9)	0.68 ***^●●●^	0.61 ***^●●●^
Cobalamin (B12)	0.37 *^●●^	0.08
Ascorbic Acid (C)	0.32 *^●●^	0.13

Spearman’s rank correlation coefficient (r_s_): * *p* < 0.05; *** *p* < 0.001. Effect Size (r): ^●●^ medium 0.11–0.49; ^●●●^ large > 0.5.

## Data Availability

The datasets presented in this article are not readily available due to their inclusion in an ongoing, larger-scale study. Request to access the datasets should be directed to ibartolomesa@upsa.es.

## Abbreviations

The following abbreviations are used in this manuscript:

MD	Mediterranean Diet
BMI	Body Mass Index
WHR	Waist to Hip Ratio
MEDAS	Mediterranean Diet Adherence Screener
IPAQ	International Physical Activity
BIA	Bioelectrical Impedance Analysis
DRI	Dietary Recommended Intake
MET	Metabolic Equivalent Task
LAG	Low-Adherence Group
HAG	High-Adherence Group
SFA	Saturated Fatty Acid
MUFA	Monounsaturated Fatty Acid
PUFA	Polyunsaturated Fatty Acid
TUFA	Total Unsaturated Fatty Acid
